# The Mechanism of Abrupt Transition between Theta and Hyper-Excitable Spiking Activity in Medial Entorhinal Cortex Layer II Stellate Cells

**DOI:** 10.1371/journal.pone.0013697

**Published:** 2010-11-04

**Authors:** Tilman Kispersky, John A. White, Horacio G. Rotstein

**Affiliations:** 1 Program in Neuroscience and Center for BioDynamics, Boston University, Boston, Massachusetts, United States of America; 2 Department of Bioengineering, University of Utah, Salt Lake City, Utah, United States of America; 3 Department of Mathematical Sciences and Center for Applied Mathematics and Statistics, New Jersey Institute of Technology, Newark, New Jersey, United States of America; University of Alberta, Canada

## Abstract

Recent studies have shown that stellate cells (SCs) of the medial entorhinal cortex become hyper-excitable in animal models of temporal lobe epilepsy. These studies have also demonstrated the existence of recurrent connections among SCs, reduced levels of recurrent inhibition in epileptic networks as compared to control ones, and comparable levels of recurrent excitation among SCs in both network types. In this work, we investigate the biophysical and dynamic mechanism of generation of the fast time scale corresponding to hyper-excitable firing and the transition between theta and fast firing frequency activity in SCs. We show that recurrently connected minimal networks of SCs exhibit abrupt, threshold-like transition between theta and hyper-excitable firing frequencies as the result of small changes in the maximal synaptic (AMPAergic) conductance. The threshold required for this transition is modulated by synaptic inhibition. Similar abrupt transition between firing frequency regimes can be observed in single, self-coupled SCs, which represent a network of recurrently coupled neurons synchronized in phase, but not in synaptically isolated SCs as the result of changes in the levels of the tonic drive. Using dynamical systems tools (phase-space analysis), we explain the dynamic mechanism underlying the genesis of the fast time scale and the abrupt transition between firing frequency regimes, their dependence on the intrinsic SC's currents and synaptic excitation. This abrupt transition is mechanistically different from others observed in similar networks with different cell types. Most notably, there is no bistability involved. ‘In vitro’ experiments using single SCs self-coupled with dynamic clamp show the abrupt transition between firing frequency regimes, and demonstrate that our theoretical predictions are not an artifact of the model. In addition, these experiments show that high-frequency firing is burst-like with a duration modulated by an M-current.

## Introduction

Information flows from the neocortex to the hippocampus through the superficial layers (II and III) of the medial entorhinal cortex (EC) [1], [2]. The spiny stellate cells (SCs) are the most abundant cell type in layer II of the medial EC, and give rise to the perforant path, the main afferent fiber system to the hippocampus [1], [3]. Previous experimental and theoretical work [4]–[11] has shown that SCs posses the intrinsic and dynamic properties that endow them with the ability to display rhythmic activity in the theta frequency range (4–10 Hz). More specifically, *in vitro* electrophysiological studies have shown that SCs display rhythmic subthreshold membrane potential oscillations (STOs) in the theta frequency range and, when the membrane is set positive to threshold, SCs fire action potentials at the peak of the STOs, but not necessarily on every STO cycle [4]. These subthreshold oscillations are intrinsic single cell phenomena [12] resulting from the interaction between a persistent sodium (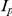) and a hyperpolarization-activated (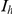) currents [4]. *In vivo* spiking patterns are similarly rhythmic in the theta range [13], although there is some question about the link between subthreshold oscillations and *in vivo* firing [14]. Theta frequency rhythmic activity in the medial temporal lobe has been implicated in learning and memory process [15]–[18] and spatial navigation [19]–[22].

SCs have been found to be hyper-excitable in animal models of temporal lobe epilepsy (TLE) [23]–[25]. In the hyper-excitable state SCs fire at a frequency much higher than theta. The proposed network mechanisms for hyper-excitability of SCs include, as their main component, a reduced level of the inhibitory inputs onto SCs in diseased animals as compared to control ones [23], [25]–[31]. Similar results to these observed in epileptic animals were shown to occur in control animals under GABA_*A*_ receptor blockade with picrotoxin [31]. A recent study [32] found evidence for the existence of (1) recurrent excitatory connections among SCs, (2) similar levels of recurrent excitation in control and epileptic animals, and (3) reduced levels of recurrent inhibition of SCs in epileptic animals as compared to control ones.

A cell's firing frequency can be described in terms of an effective time-scale operating in the subthreshold regime and resulting from current balances occuring there. For SCs in the theta frequency range, 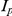 activates fast and provides the main drive for the depolarization phase of STOs while 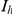 (hyperpolarization-activated with slow kinetics) provides a delayed feedback effect that promotes resonance. In an isolated cell, the spikes occurring at the peak of STOs are the result of imbalances among 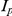, 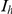 and the tonic drive (constant applied current) [4], [10], [11], [33]. Each subsequent spike will occur after roughly a theta cycle. Theoretical studies [8] have shown that spiking at theta frequencies persists in recurrently connected SCs for significant levels of synaptic AMPA excitation, and SCs synchronize in phase. We reasoned that, dynamically such a network behaves as a single, self connected SC. Accordingly, a synaptic pulse of AMPA excitation arrives immediately after a spike has occurred. The initial SC's fast depolarization provides a window of opportunity for a new spike to occur (shortly after the previous one) provided the amount of excitatory current is large enough to overcome the combined effect of the theta pacemaking currents before they fully develop (note that EPSPs activate 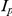 but deactivate 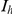). This amount of excitatory current depends on the the maximal synaptic conductance and the synaptic decay time. The interspike interval (ISI) of two such consecutive spikes would be much shorter than a theta cycle and would constitute an effective fast time-scale for the autaptically connected SCs and hence for recurrently connected network of SCs. We hypothesized that under biophysically plausible conditions recurrently connected networks of SCs are able to fire in these two well separated frequency regimes depending on the levels of recurrent synaptic excitation but not at frequencies in between. The fast frequency regime corresponds to hyper-excitable firing. In the context of this paper we use term hyper-excitability as the ability of the SC to fire in this fast frequency regime.

In this paper we investigate this hypothesis and the underlying biophysical and dynamic mechanism that leads to hyper-excitable firing in SCs, including the role played by synaptic inhibition (opposing the effects of synaptic excitation) and the mechanism of termination of fast frequency spiking leading to hyper-excitable bursts similar to the ones observed experimentally [31]. In our investigation we combine modeling, simulations, dynamical systems tools and dynamic clamp experiments.

## Results

We use a biophysical (conductance-based) model for SCs that has previously been used to investigate several aspects of SC dynamics including the mechanism of generation of rhythmic activity at theta frequencies (subthreshold oscillations and spikes) in single SCs and recurrently connected networks including SCs and interneurons [8], [10], [11]. This model is based on measurements from layer II SCs [4], [33]–[35], and it includes a persistent sodium (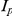), a two-component (fast and slow) hyperpolarization-activated (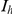 or h-) current, and a slow potassium (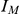 or M-) current. The model is described in the Methods section. We first show that minimal model networks including SCs and interneurons (ICs) exhibit a transition between the tw firing frequency regimes (theta and hyper-excitable) as the result of reduced levels of inhibition onto SCs, thus capturing a main feature of the results by Kumar et al. [31]. We then show that this transition also occurs for recurrently connected SCs in the absence of inhibition and that it is abrupt (threshold-like); i.e., the constant theta firing frequency maintained for significant levels of recurrent excitation [8] breaks at a threshold level and a sudden increase in firing frequency occurs. We observe similar transitions in single SCs self-connected with an autapse. However, this transition does not occur in isolated SCs by increasing tonic (constant) excitation. Next, we use phase-space analysis to provide a geometric/dynamic explanation of these results. More specifically, we explain how 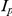 and 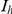 interact with AMPA synaptic excitation to produce the two modes of firing frequency operation for sub- and super-threshold levels of recurrent excitation. By using this approach we demonstrate that the fast time scale, just like the theta time scale, is “built in” the SC model but is occluded in isolated cells and uncovered by phasic excitation. Differently from other mechanisms for similar phenomena [36], we have not observed bistability between the corresponding firing frequency regimes. Experiments using dynamic clamp show that real SCs exhibit abrupt transition between firing frequency regimes; i.e., our theoretical findings are not an artifact of the model. Finally, the combination of this experimental approach and simulations using single cells uncovers a new role for the M-current in modulating the duration of bursts of hyper-excitable activity.

### Minimal networks capture hyper-excitable behavior

#### Hyper-excitability in recurrently connected stellate cells with reduced inhibition

**Figure 1:**
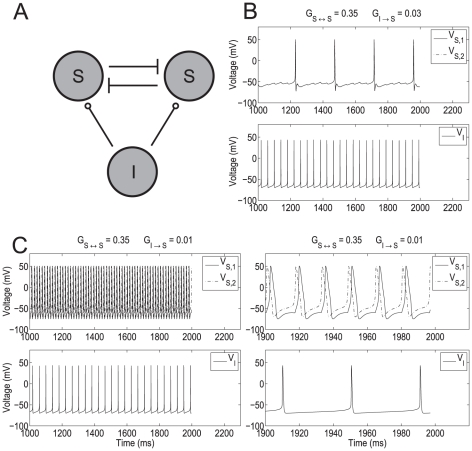
Abrupt transition from the theta to the hyper-excitable regime in a SI minimal network. **A**: Diagram of the minimal SI network. Stellate cells (S) excite each other (AMPA) and receive inhibition (GABA_*A*_) from an interneuron (I). The maximal synaptic conductances 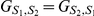 and 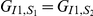 are represented by 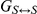 and 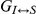 respectively. **B** and **C**: show the transition from the theta to the persistent hyper-excitable regime as the result of a small decrease in inhibition. In the theta regime SCs are synchronized in phase while in the hyper-excitable regime SCs are synchronized slightly out of phase. The right panel in **C** is a magnification of the left one. The parameters used are 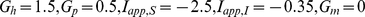, and 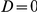.

**Figure 2:**
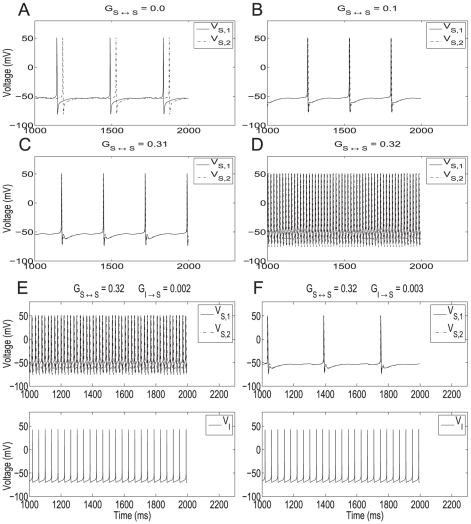
The role of synaptic excitation and inhibition in the abrupt transition from the theta to the hyper-excitable regimes in two recurrently connected SCs. **A–D**: Abrupt transition from the theta to the hyper-excitable regime in two recurrently connected SCs as a result of a small increase in the amount of excitation. **A**: In the absence of recurrent excitation the SCs fire out of phase. **B** and **C**: Recurrent excitation synchronizes the SCs in phase but the firing frequency remains almost unchanged in the theta regime. **D**: A small increase in the maximal synaptic conductance causes the abrupt transition to the hyper-excitable regime. The parameters used are 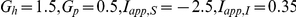. **E and F**: A small increase in inhibition to the two recurrently connected SCs reverses the firing frequency from the hyper-excitable to the theta regime. The parameters used are 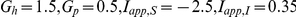.

**Figure 3:**
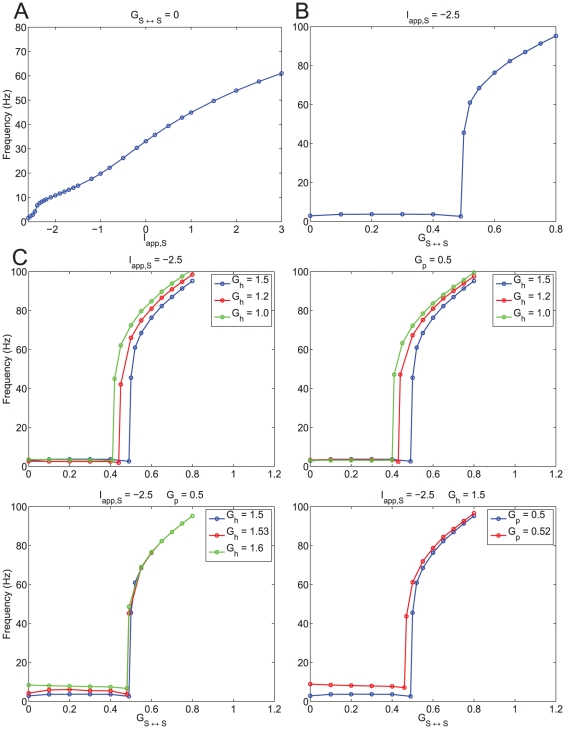
The abrupt transition from the theta to the hyper-excitable regime is the result of phasic but not tonic excitation. **A**: Firing frequency of a single isolated SC as a function of the applied DC current 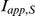. The transition from low to high frequencies is smooth. **B**: Firing frequency of a single SC self-excited via an autapse as a funciton of the autapse maximal synaptic conductance 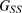 (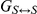). The transition from low to high frequencies is abrupt. **C**: Effects of persistent sodium and h-currents on hyperexcitability in a self-connected single SC. Top-left panel: A decrease in the h-current maximal conductance 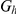 facilitates hyperexcitability for a fixed applied DC current 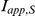. Appropriate values of 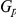 were chosen in order to obtain close values of the SC's firing frequency for for 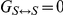 (isolated cell). Top-right panel: A decrease in the h-current maximal conductance 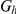 facilitates hyperexcitability for a fixed maximal persistent sodium conductance 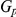. Appropriate values of 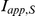 were chosen in order to obtain close values of the SC's firing frequency for for 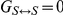 (isolated cell). Bottom-left panel: Changes in the h-current maximal conductance 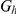 have little effect on hyperexcitability for fixed values of 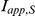 and 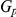. Bottom-right panel: An increase in the amount of persistent sodium facilitates hyperexcitabilit for fixed values of 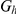 and 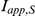. In all cases, simulations were performed using the (7D) “full” SC model.

We used a minimal network model consisting of two stellate cells (SCs) and one interneuron (IC) (Figure 1-A) to capture the transition between firing frequency regimes as the result of reduced levels of inhibition onto SCs. The two SCs are connected via excitatory AMPAergic synapses. Each of these cells receives GABAergic inhibition (GABA_*A*_) from the IC. In Figures 1, 2 and 3, the maximal synaptic conductances between the two stellate cells are equal (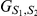 = 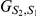) and represented by 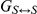. The inhibition to both SCs is also equal (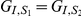) and is represented by 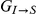.

Figure 1 shows representative examples of the transition from normal (theta) to hyper-excitable spiking activity in SCs resulting from a decrease in synaptic inhibition. In these figures, the natural frequency of each SC (the firing frequency of the cell in the absence of any excitatory and inhibitory input) was set at 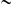
3 Hz with an underlying subthreshold oscillation frequency approximately equal to 9 Hz. This produces a regular pattern of three subthreshold oscillations per spike (Figure 2-A). In the absence of any synaptic input the two SCs fire with a relative phase that depends on the initial conditions. The natural frequency of the IC was set to 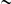
25 Hz [37]. During normal activity (Figure 1-B), the firing frequency of the synchronized in phase coupled SCs (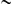
4 Hz) is slightly higher than the uncoupled ones (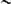
3 Hz). The higher firing frequency is due to excitation combined with repeated inhibitory pulses that result in activation of the h-current [10], [11], [38] producing a net depolarizing effect. Figures 1-B and -C differ only in the value of 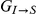, which is lower in Figure 1-C and represents the decreased inhibition seen in epileptic rats [31]. For larger values of 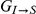 (Figure 1-B), SCs fire in the theta regime at 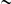
4 Hz and synchronize in phase as occurs for recurrently connected networks of SCs in the absence of inhibition [8]. When 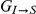 is reduced (Figure 1-C), SCs fire at 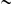
60 Hz and synchronize slightly out of phase (Figure 1-C, top-right panel).

#### A small increase in the SC recurrent synaptic conductance causes an abrupt increase in the SC's firing frequency in the absence of inhibition

In order to understand the role of recurrent excitation in the abrupt transition between firing frequency regimes, we modified the minimal model network considering only two synaptically connected SCs with no inhibition (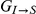 = 0). Figure 2-A shows voltage traces of two uncoupled SCs (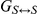 = 0). In this case, the phase relationship of the cells depends only on their initial conditions. When coupling is added (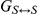 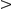
0), Figures 2-B and -C, SCs synchronize in phase in the theta frequency regime as previously shown in [8]. Notably, in Figures 2-B and -C, which differ significantly in the strength of 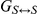 coupling, SCs fire at nearly the same frequency showing that firing in the theta frequency regime is robust to changes in the levels of recurrent excitation. However, when 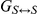 is further increased by a small amount an abrupt transition in the firing frequency occurs (Figure 2-D). This large change in the SC's firing frequency for a small change in the maximal conductance 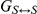 has the characteristics of a threshold-like phenomenon occurring for a value, or a very narrow range of values, of 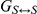 in between 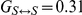 and 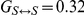 (Figures 2-C and -D).

### A small increase in the inhibitory input to fast firing SCs abruptly reverses their firing frequency to the theta regime

Here we show that the inhibition acts as a switch between firing frequency regimes. That is, inhibition uncovers the fast time scale created by recurrent excitation in the minimal network model but is not necessarily involved in its generation. To this end, we reintroduce a small amount of inhibition to the network of two coupled SCs (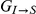 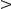
0). We started with the network firing in the hyper-excitable regime (parameters like those in Figure 2-D). Figures 2-E and -F show that a small increase in inhibition can abruptly cause a transition back to the theta state. The value of 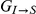 at which the transition to hyper-excitable firing occurs is dependent on the chosen value of 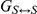 and, in the representative example shown, lies between the two values used in Figures 2-E and -F. No bistability between firing frequency regimes was observed in this inhibition-containing network just as no such bistability was observed in the network containing recurrent excitation. We discuss this point further in the next sections.

#### The abrupt changes in the SC firing frequency are the result of phasic but not tonic excitation

Here we show that the abrupt transitions between the theta and hyper-excitable firing frequency regimes observed in recurrently connected networks of SCs do not occur in single, isolated SCs. To simplify our study, we followed other authors [36], [39], [40] and studied a single SC synaptically connected to itself with an “autapse”. We justify this approximation by noting that model SCs synchronize in phase in the theta regime and very close to in phase in the hyper-excitable regime This approximation lends itself well to later experimental and analytical investigation.

We compared the effects of increasing tonic (constant) excitation (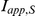) in the isolated cell (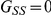) with increasing phasic excitation, via increasing the maximal synaptic conductance 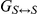, in an autaptically coupled model SC for constant values of the tonic drive. Figure 3-A shows a graph of frequency as a function of 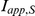 for 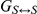 = 0 (only tonic excitation). In this regime the SC can reach high spike rates with no abrupt changes in the firing frequency. Note that although the SC can fire at frequencies corresponding to the hyper-excitable regime, the change in the amount of 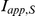 needed for the transition from the theta regime seems biophysically implausible.

Figure 3-B shows a graph of frequency as a function of 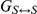 for a constant value of the tonic drive 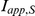 (only changes in phasic excitation). The constant value of 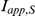 chosen is equal to the one used in the previous sections. Figure 3-B shows an abrupt jump in the firing frequency occurring in a very small range of values of 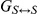. Simulations for values of 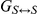 in this range shows spiking burts with fast intra-burst spiking frequency (corresponding to the hyper-excitable regime). They are reminiscent of the bursts observed in the presence of an M-current (discussed later, see also Figure S7-b). For the remainder of this paper we will approximate this narrow range by a threshold value. As a consequence of the approximation (single, self-connected SCs representing a recurrently connected SC population), the threshold values for hyper-excitability are higher than the ones found for the two recurrently connected SCs. Qualitatively, however, these simulation results show that increasing phasic excitation (by increasing maximal autapse strength, 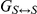) past a threshold value causes a rapid transition to the hyper-excitable regime (Figure 3-B). Voltage traces illustrating this transition are presented in Figure S7-a.

Further simulations show that there is no histeresis, and hence no bistability, between firing frequency regimes as the result of increasing 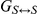. (See also Figure S2 and Supporting Text S1) Lack of bistability often occurs in nonlinear systems showing abrupt transitions between disparate dynamic regimes. A typical example is the abrupt transition in the amplitude of limit cycles created in a super-critical Hopf bifurcation due to the canard phenomenon in two-dimensional fast-slow systems such as the FitzHugh-Nagumo model [41], [42]. Note that in the model we use (see Methods) the transition from subthreshold oscillations to spikes in isolated cells also occurs via a canard mechanism [10], [11].

The dynamic mechanism we describe here is different from previous work on recurrently connected excitatory networks. AMPA recurrent excitation has been shown to lead to high frequency firing in networks of integrate-and-fire (I-F) neurons [36]. In these models there is bistability between a rest state and a high frequency spiking state, and the minimal firing rate increases as 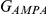 (our 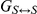) increases. In addition, in I-F models the frequency of isolated cells increases with increasing amounts of both phasic and tonic excitation. In contrast, we show that SCs recurrently connected via AMPA synaptic excitation display spiking activity in two frequency regimes (theta and hyper-excitable) with no bistability between them. In addition, the SC's firing frequency remains almost constant until the levels of AMPA excitation reach the threshold for the abrupt transition; i.e., the SC's firing frequency in the theta regime is very robust below the threshold for hyper-excitability. Moreover, the model we use, unlike I-F models, contain biophysical information about SCs that allow for the investigation of the effect of the cell's intrinsic currents on hyper-excitabiliy. Finally, in the recurrently connected networks considered in [36], AMPA receptor mediated synaptic excitation produced firing frequencies much higher than the ones corresponding to our hyper-excitable regime. Lower firing frequencies required either NMDA receptor mediated synaptic excitation or synaptic depression mechanisms [36]. Note that due to its slow decay time NMDA currents are almost constant for the time scales corrsponding to the hyper-excitable regime and thus would produce no qualitative change in the behaviors described in this paper. Hyper-excitable firing due to recurrent synaptic excitatory connections and controlled by synaptic inhibition has been previously shown to occur in other areas of the hippocampal formation such as hippocampal region CA3 [43]–[46]. However, the set of intrinsic currents present in CA3 pyramidal cells is different from these controlling the dynamics of SCs in the subthreshold regime. Synaptic excitation with slow decay times has been shown to significantly reduce the firing frequency in networks of Hodgkin-Huxley (HH) neurons [40] leading to low firing frequency patterns consisting of spikes interspersed with subhtreshold oscillations at much higher frequencies. Although this scenario has some similarities with the one displayed by SCs in the low firing frequency regime, the two are qualitatively different. First, in both cases the corresponding slow time scales are generated by a three-dimensional canard mechanism [10], [11], [40], [47]. However, for SCs the slow time scale is generated by the intrinsic currents (mostly 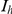 and 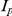) with no participation of synaptic excitation while synaptic excitation is necessary for the networks of HH neurons considered in [40]. In addition, as we showed above, the SC's firing frequency is maintained for significant levels of recurrent excitation before the abrupt transition to high-frequency firing occurs while for the HH neurons in [40] firing firing frequency decreases with stronger synaptic coupling. Finally, for SCs subthreshold oscillations occur at theta frequencies and result from the interaction between 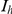 and 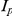 with no participation of the spiking currents (transient sodium, 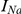, and delayed-rectifier potassium, 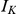) while for networks of HH neurons subthreshold oscillations have a frequency much higher than theta and result from the interaction among synaptic excitation, 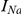 and 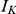.

#### The effect of the SC's intrinsic currents on abrupt transition between firing regimes

The abrupt transition between firing frequency regimes persists upon changes in the SC's intrinsic currents 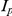, 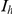 and the tonic drive 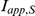. The effect of these changes are reflected mostly in the threshold values for 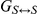. In most cases we compared among parameter sets where the levels of 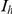, 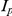 and 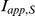 are balanced so that the resulting baseline frequency in the theta regime is almost unchanged (see Figure S3). Our results are presented in Figure 3-C. (See also Figure S2 and Supporting Text S1). Figure 3-C (top panels) show that for a given baseline frequency (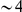 Hz) in the theta regime, the threshold for the abrupt transition increases with 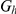. The top-left panel corresponds to a constant value of 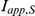 and pairs of balanced values of 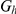 and 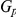 while the top-right panel corresponds to a constant value of 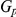 and pairs of balanced values of 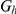 and 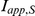. In Figure 3-C (bottom-left panel) both 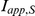 and 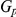 are constant, and consequently the baseline theta frequency increases with 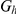 (see also Figure S3) but the threshold for the abrupt transition remains almost constant. Figure 3-C (bottom-right panel) corresponds to constant values of 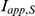 and 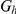. The baseline theta frequency increases with 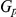 but the threshold for the abrupt transition decreases with increasing values of 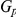. The effect of changes in 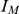 will be considered later in the paper.

### The dynamic mechanism of abrupt transition between firing frequency regimes due to phasic excitation

Phase-space analysis is a useful geometric tool for building a qualitative understanding of the evolution of dynamical systems. Here we use dynamical systems tools to provide a geometric/dynamic explanation of the mechanism underlying the abrupt transition between firing frequency regimes in a single model SC, self-connected via AMPA excitation. In particular, we show that the two time scales (theta and fast) are built into the isolated SC, explain their dependence on the model parameters, in particular 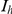, 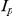 and 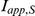, and show how these currents interact with synaptic excitation.

We extend the nonlinear artificially spiking (NAS) SC model derived in [10] to include synaptic currents (see Methods for the description of this model for an isolated SC). NAS models include integrate- and resonate-and-fire neurons. The NAS-SC model consist of a set of three equations describing the dynamics of the the voltage 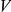 and the two 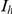 gating variables, (fast 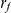 and slow 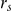) in the subthreshold voltage regime, a voltage threshold 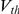 indicating the occurrence of a spike and a reset value for the dynamic variables after a spike has occurred. This model captures the main aspects of the dynamics of the “full” (7D) SC model in the subthreshold regime [10] (see Methods): the generation of subthreshold oscillations and the onset of spikes. The model also includes 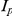, slaved to 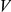 due to the fast dynamics of its associated gating variable 
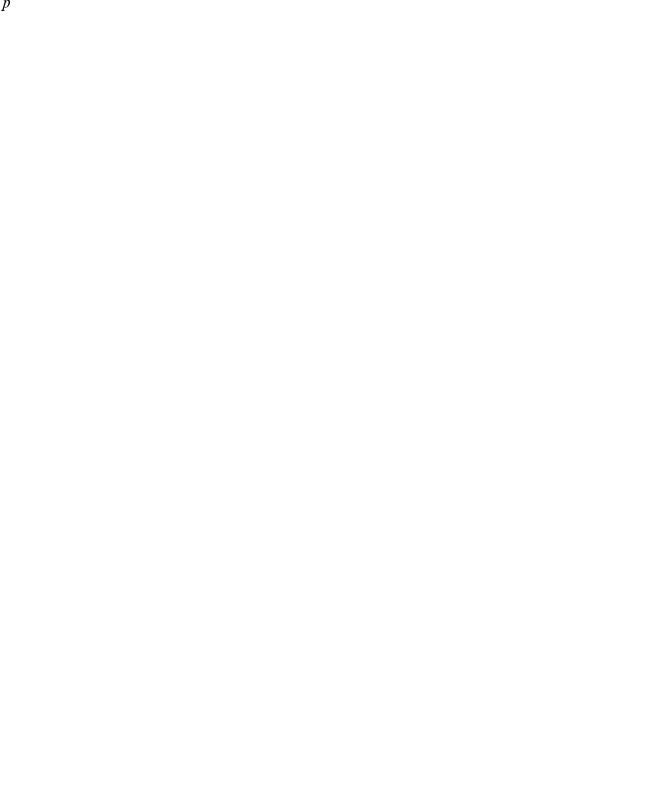
, but it does not include the spiking currents 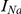 and 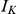, which were found to be negligible in the subthreshold regime [10], nor 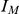 which, as suggested by other authors [48] and confirmed by our experiments (described later in this paper), is not active at subthreshold voltage levels and does not alter the low frequency firing behavior.

In order to account for the effect of synaptic excitation, we extend the reduced NAS-SC model Eqs. (14)–(16) described in Methods for a single SC to include a synaptic current term (autapse) 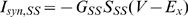 in the voltage equation, and a fourth equation describing the evolution of the synaptic variable 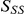. The resulting equations are:
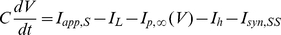
(1)

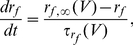
(2)

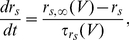
(3)

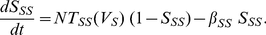
(4)


#### The (reduced) NAS-SC model captures the abrupt changes in the firing frequency due to phasic self-excitation

**Figure 4:**
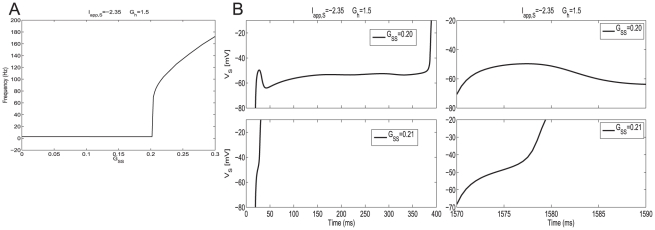
The NAS-SC model self-connected with an autpase captures the abrupt transition between the theta and hyper-excitable regimes as the result of small changes in the maximal synaptic conductance 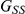. **A**: Spiking frequency vs. 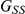 for representative values of 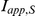 and 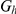. The spiking frequency is measured in number of spikes per second. The transition point corresponds to 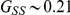. **B**: Voltage traces for one representative values of 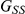 on each side of (and close to) the transition point. The value of the persistent sodium maximal conductance is 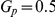. The interspike intervals are 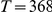 ms (top-left) and 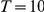 ms (bottom-left). The right panels are magnifications of the left ones.

In Figure 4-A we show a graph of firing frequency vs. 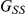 for representative values of 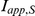, 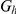 and 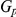. For these parameter values there is a jump in firing frequency for 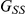 in a range of values in the interval 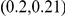. Figure 4-B shows the voltage traces for 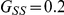 (top panels) and 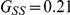 (bottom panels), on each side of, and close to the transition values. Time 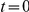 corresponds to the occurrence of a spike and hence the initiation of the EPSP. The total period for the unperturbed SC is 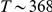 ms. The interspike-interval decreases from 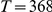 ms (Figure 4-B, top-left panel) to 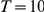 ms (Figure 4-B, bottom-left panel). Comparison between the top and bottom panels shows that in both cases, the voltage first depolarizes fast at the beginning of the interspike interval and then it continues to depolarize fast for 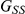 = 0.21, eventually spiking, while for 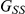 = 0.2 the trajectory “turns around” and hyperpolarizes, displaying a “bump” and continues to evolve on a much slower time scale. Note that the bump period is much smaller than the subthreshold oscillation period, and of the same order of magnitude as the fast spike. Qualitatively similar results are obtained for other combinations of parameters indicating this behavior is robust (data not shown). Note that the transition values of 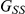 are lower than the ones corresponding to the full model. This is due to the NAS-SC model approximation described above. However, consistent with the full SC model, transition values are the same as those observed for two recurrently coupled NAS-SC cells initially in phase (data not shown).

#### The dynamics of isolated SCs: Phase-space analysis, nonlinearities and time-scale separation

**Figure 5:**
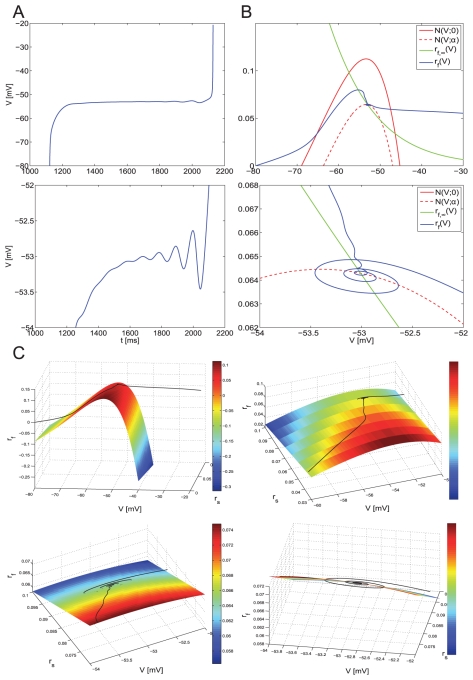
Phase-space diagram for the NAS-SC model in the theta regime (slow time scale). Trajectories begin evolving from reset values 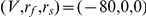 (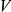 measured in mV). **A**: Voltage trace corresponding to the trajectory shown in **B** and **C**:. The bottom panel is a magnification of the top one and shows subthreshold oscillations. **B**: Two-dimensional projection of the phase-space diagram shown in **C**. The two curves in red are contained in the V-nullsurface. The bottom panel is a magnification of the top one. **C**: For different views of the 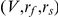 phase-space diagram showing only the V-nullsurface and the trajectory. The values of the parameters are 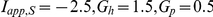.

The NAS-SC model for the isolated SC (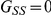) is three-dimensional and is described by Eqs. (1)–(3). (The equation for 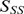 is decoupled from system (8)–(16).) Figure 5-A shows the trace of one firing period generated by the NAS-SC model for a biophysically plausible set of parameters (and 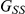 = 0). The initial conditions were set at 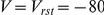 and 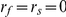 which approximate the reset values of the variables after a spike has occurred. Importantly, when capturing the SC's dynamics after a spike has occured, not all initial conditions in the subthreshold regime are possible but only these reset values (see Methods and [10]). In Figure 5-A, the voltage first increases fast, then evolves on a slower time scale displaying a few subthreshold oscillations (Figure 5-A, bottom panel), and finally increases fast marking the initiation of a spike. (Spikes are the result of the activation of 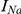, which is not described by the NAS-SC model and belongs to the spiking regime as explained in Methods.)

Figures 5-B and -C show the phase-space corresponding to Figure 5-A. Trajectories join the points 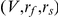 corresponding to different values of 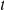. As 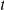 increases, the tip of the trajectory moves describing the evolution of the dynamical system (1)–(3). The set of points resulting from setting the right hand sides of Eqs. (1)–(3) equal to zero define surfaces, called nullsurfaces, on which there is no motion in the direction of the corresponding variable. Nullsurfaces, and their shapes, play an important role in the description of a phase-space. The 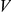-nullsurface for system (1)–(3) with 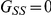, is given by
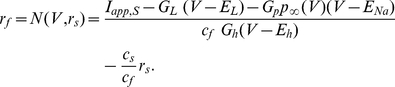
(5)


The 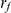
- and 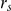
- nullsurfaces are given by 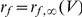 and 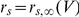 respectively. The speed of the trajectory is not captured by the phase-space. Additional information has to be taken either from the voltage traces or from the parameter values. The SC model in the subthreshold regime is a fast-slow system. 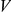 is the fast variable and both 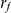 and 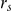 are the slow variables; i.e., both 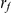 and 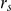 evolve on a much slower time scale than 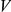. This time-scale separation is crucial for the phenomenon we are explaining here. In Methods we show how these time scales are uncovered by bringing the system (1)–(3) to the dimensionless form.

Trajectories evolve on a combination of both time scales: fast along horizontal directions and slow on small neighborhoods of the V-nullsurface. Figure 5-C shows the V-nullsurface 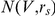 and the trajectory corresponding to Figure 5-A. This trajectory moves fast from its initial point 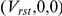 towards the left branch of the V-nullsurface (Figure 5-C, top-left panel) (fast voltage increases) and then slow in a vicinity of the 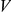-nullsurface (the so called slow manifold, along which 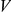 increases slow) towards the curve of knees (Figure 5-C, top-right panel). Once trajectories reach the curve of knees, they oscillate (Figure 5, bottom panels) [10], and finally move fast in the direction of increasing values of V. This unbounded motion in the horizontal direction signifies that the trajectory escapes the subthreshold regime and moves into the spiking regime where the dynamics are governed by a different set of currents. Due to the nature of the system's dynamics (vector field), once a trajectory starts moving along this fast horizontal direction it cannot stop unless externally forced. Thus, once a trajectory is on the right side of the V-nullsurface it will spike. We emphaize that spiking is indicated, but not described, by 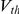.

Figure 5-B shows a compact (two-dimensional) description of the phase-space where the two-dimensional 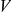
- and 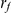-nullsurfaces are projected onto the 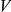-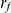 plane. The top panel shows the curves 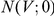 and 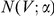 (for some 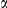 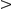
0), which are the result of the intersection of 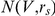 with constant planes 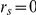 and 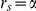 respectively. Both curves are contained in the nullsurface 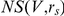 given by (5). Note that as 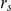 increases, the V-nullsurface decreases; i.e., the curve of knees joining the maxima of the curves 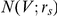 is a decreasing function of 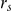. In the following sections we will use this compact description.

From our previous discussion, the evolution of the trajectory describing the state of the system at different times within an interspike-interval (ISI) can be qualitatively understood by looking at the nullsurfaces, in particular the 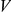-nullsurface, and the time-scale separation between the participating variables which separates the phase-space into regions of fast and slow motion. The 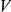-nullsurface and the relative time scales are the main components of the system's dynamic structure. They convey information about 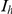, 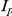 and 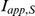, and relations among them through Eq. (5), defining the 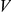-nullsurface, the non-dimensionalization results in the Methods section, and the Figure S1. Due to the presence of two time scales, trajectories spend most of the interspike interval (spiking period) in a vicinity of the 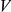-nullsurface. Consequently, the SC's interspike interval (spiking period) can be approximated by the time the trajectory spends in a neighborhood of the V-nullsurface. Thus, the nullsurface represents the balance among 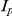, 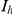 and 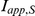 necessary for the cell to fire at a specific frequency. An increase in 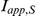 favoring spiking at a higher frequency is reflected in a “lower” 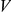-nullsurface. For instance, the maximum of the V-nullsurface corresponding to 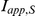 = −2.35 (Figure S4-b) is lower than that corresponding to 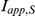 = −2.5 (Figure 5-B) reflecting the fact that, as the applied current increases the time the trajectory spends in the subthreshold regime decreases and thus the firing rate increases (Figure S4-a). Changes in other parameter values such as 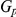 and 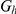 have analogous effects on both nullsurfaces and firing frequency. Figure S3 summarizes some of these effects.

By looking at the effect that changes in the biophysical parameter values have on the 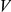-nullsurface and time-scale separation one can predict how these changes will affect the cell's dynanics. This approach has been useful in the investigation of the mechanism of generation of subthreshold and spikes in the SC model, and in particular about the role of 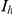, 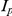 and 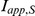 in the generation of the theta time scale through equation (5) and the results in [10], [11]. We refer the reader to these papers for further details.

#### Gradual (non-abrupt) increase in firing frequency due to tonic excitation

**Figure 6:**
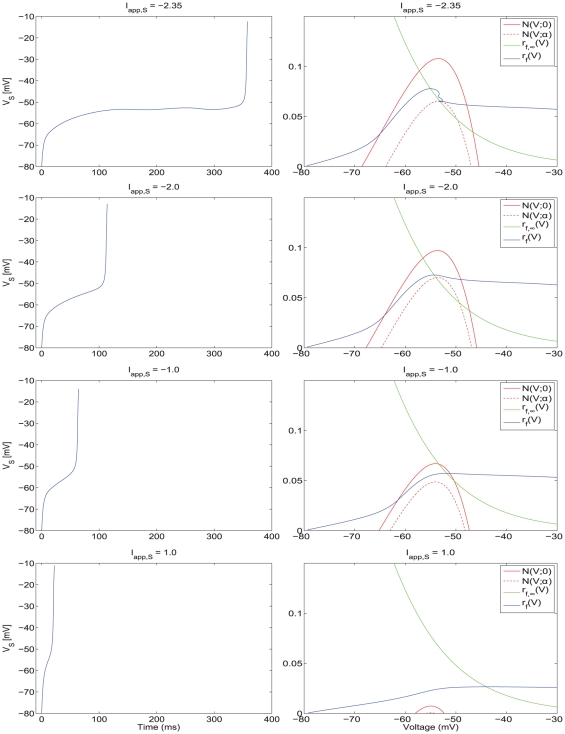
Voltage traces and two-dimensional phase-space representations for the NAS-SC model for increasing values of 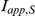 **.** Each row shows the voltage trace (left) for a particular value of 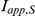 and the corresponding phase space diagram (right). As the value of 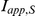 gradually increases (top row lowest value of 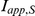, bottom row highest value of 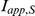, the V-nullsurface gradually moves down causing the firing frequency to increase. As the model is not self-connected in this case, increasing 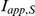 causes a gradual increase in firing rate proportional to the applied current. Increasing values of 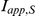 cause a progressive decrease in the influence of the slow manifold on the trajectory and thus no rapid transitions in firing rate are observed.

In Figure 6, and in more detail in Figures S5 and S6, we present the voltage traces (left panels) and phase-space diagrams (right panels) for various values of 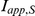 increasing from top to bottom. As 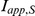 increases, the nullsurfaces move down by an amount almost proportional to the value of 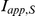, and the region of the slow manifold available for the trajectory to move along decreases. As a consequence, the time the trajectory spends on the slow manifold decreases almost linearly with the value of 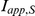 and the cell's firing frequency increases nearly linearly with the value of 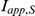. The cell's highest firing frequency corresponds to the trajectory moving along the fast (horizontal) direction without being “captured” by the slow-manifold (Figure 6, bottom panel). Since the displacement of the V-nullsurface as a consequence of increasing values of 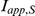 is gradual, so is the transition from the lower (theta) to higher (hyper-excitable) firing frequencies. Thus, there are no abrupt changes in firing frequency due to increases in tonic excitation.

For each choice of parameters, in particular for each choice of the tonic drive 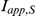, the NAS-SC model has a unique interspike-interval (a unique spiking period) which is determined by the parameter values and the initial condition 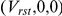 which, again, reflects the fact that 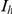 resets after a spike has occured. Consider parameter values corresponding to Figure 5 for which the SC fires in the slow (theta) frequency regime. Hypothetically, for the SC to fire also in the fast frequency regime for this same set of parameter values, the initial conditions should be located above the level of knee of the 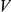-nullsurface in phase-space; i.e., initially, 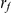 should be higher than the maximum of the 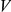-nullsurface. If this happened, then the trajectory would evolve along a fast, horizontal direction thus reaching the end of the subthreshold voltage range in a short time interval corresponding to fast spiking. (Note that this condition is equivalent to requiring 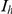 to be initially large enough to disrupt the current balance corresponding to slow spiking.) However, as we mentioned earlier, the initial conditions in the subthreshold regime are not arbitrary but correspond to the reset values after a spike has occurred and are “dictated” by the “full” (7D) model [10]. Trajectories starting these initial conditions are “captured” by the 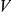-nullsurface and forced to evolve (at a slower pace) in its vicinity until reaching the knee.

#### Abrupt increase in firing frequency due to phasic excitation

Due to the addition of the synaptic variable 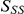, system (1)–(4) is four-dimensional. Geometrically, the generalized nullsurfaces (the set of points that make the right hand side of equation (1) equal to zero) are three-dimensional objects:
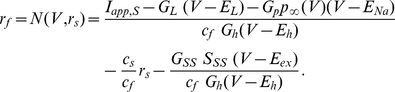
(6)


**Figure 7:**
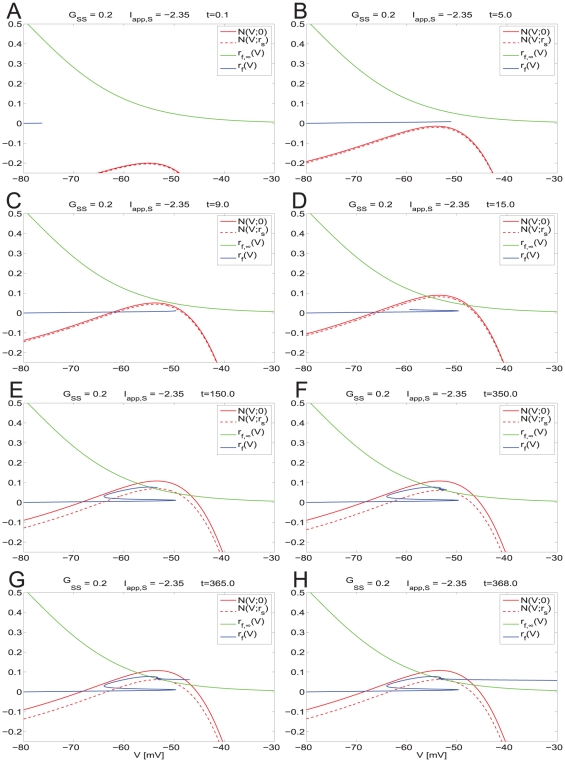
Dynamic two-dimensional phase-space representation of one firing phase for the self-connected NAS-SC model in the theta regime. Parameter values are 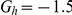 and 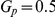 and fixed for all diagrams. The value of 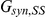 is below the threshold for hyper-excitable firing and thus a theta frequency ISI is observed. Each panel shows the evolution of the phase space for time points through one firing phase. Time increases from **A** to **H**. As the trajectory evolves it is captured by the slow-manifold (vicinity of the V-nullsurface) and is forced to move around it on a slow time scale thus causing subthreshold oscillations.

**Figure 8:**
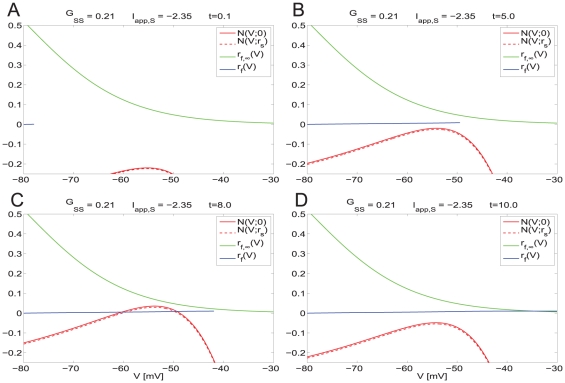
Dynamic two-dimensional phase-space representation of one firing phase for the self-connected NAS-SC model in the hyper-excitable regime. Parameter values are as in Figure 7 except that the value of 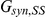 is above the threshold for hyper-excitable firing and thus a very short ISI is observed. Each panel shows the evolution of the phase space for time points through one firing phase. Time increases from **A** to **D**. The trajectory evolves along the fast time scale and it manages to escape the subthreshold regime without being captured by the slow manifold (vicinity of the V-nullsurface).

The corresponding phase-space diagram can be visualized by extending the previous approach used for the isolated NAS-SC model to include values of 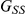 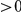. Figure 7 corresponds to the traces shown in Figure 4-B (top panel) where the SC fires in the theta regime. Figure 8 corresponds to the traces shown in Figure 4-B (bottom panel) in which the SC fires in the fast (hyper-excitable) regime. The initial conditions in both cases (Figures 7 and 8) correspond to the reset values of the trajectory after a spike has occurred. Note that the synaptic variable 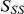 is parameterized by time; thus the 3D generalized V-nullsurface can be seen as a two-dimensional 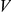-nullsurface moving as time evolves. More precisely, in each panel we plot the V-nullsurface corresponding to a representative value of 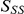
(t). After a spike has occurred, the synaptic variable 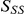 first increases and then decreases with the time scales corresponding to the rise (fast) and decay (slow) times of AMPAergic excitation. We use the parameters in [8]. As a consequence, the V-nullsurface (6) first moves down (fast) (Figure 7-A) until 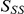 has reached its maximum. When 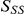 starts decreasing, the V-nullsurface moves back up on a slower time scale (Figure 7-B) corresponding to the AMPA decay time. Given a specific firing frequency in the theta (slow) frequency regime. For each value of 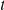, and hence for each value of 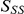, the distance between the peaks of the unperturbed and perturbed 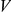-nullsurfaces measures the instantaneous magnitude of the current balance disruption due to synaptic excitation. The current balance is recovered when the 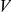-nullsurface returns to its original position (or close to it).

If the trajectory moving along the horizontal direction is slow compared with the speed at which the V-nullsurface moves up, then it may be caught inside it (Figure 7-C). If this occurs, then the trajectory is forced to reverse its direction and move towards the left (Figure 7-D). This change in the trajectory's 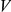 direction of motion gives rise to the fast frequency “bump” observed in Figure 5-B (top panel). After crossing the left branch of the V-nullsurface the trajectory moves along the slow manifold (small neighborhood of the V-nullsurface) (Figure 7-E). When it reaches the knee (Figure 7-F) it oscillates eventually escaping the subthreshold regime firing an action potential (Figures 7-G and -H). As before, since all other processes occur along fast directions, the cell's spiking period is determined by the time the trajectory spends in the vicinity of the slow manifold (vicinity of the V-nullsurface). Thus, the spiking period of the coupled (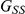 = 0.2) and uncoupled (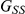 = 0) cases do not differ by much. In this case, the SC's current balance recovered fast enough to prevent the new spike from occurring right after the previous one.

This geometric picture changes for 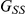 = 0.21 (Figure 8). For this value of 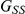, the V-nullsurface does not move upwards fast enough to be able to capture the trajectory inside it (Figure 8-C). As a consequence, the trajectory is able to keep moving along the fast (almost horizontal) direction and escapes the subthreshold regime, firing an action potential, without being influenced by the slow manifold. The SC's spiking period and firing frequency are thus determined by the time it takes the trajectory to traverse the subthreshold regime (fast time scale) leading to hyper-excitability. In this case, the SC's current balance did not recover fast enough to prevent a spike to occur right after the previous one. In the absence of any change in the model parameters these dynamics repeat and lead to persistent firing in the fast frequency regime.

Taken together, these results and the results of the previous sections explain the genesis of the two time-scales present in SCs (theta, slow and hyper-excitable, fast) and the abrupt transition between them as the result of interactions between the SC's intrinsic currents and recurrent phasic (AMPA) excitation. Additionally, our results show the role that AMPA synaptic kinetics play in this process.

### Dynamic clamp experiments confirm theoretical findings and suggest a novel role for the M-current in stellate cells

#### *In vitro* SCs undergo abrupt transitions in firing rate due to small increases in self-excitation

**Figure 9:**
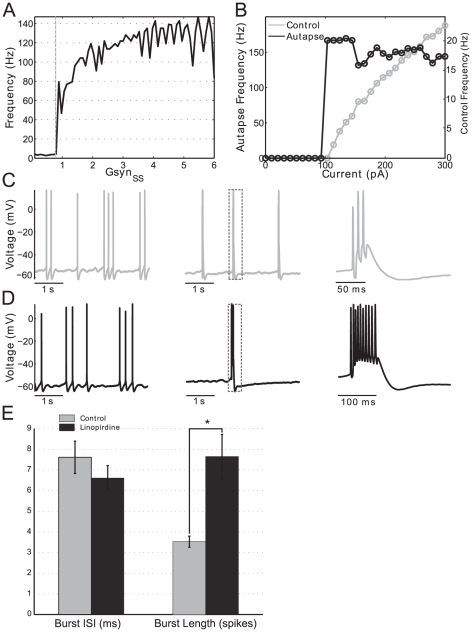
Sudden transitions from regular spiking to burst firing in EC stellate cells. **A**: Instantaneous frequency (the frequency corresponding to a single ISI, see Methods) jumps rapidly in autapse ramp experiments. In this representative example, autapse strength (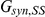) was ramped up gradually exposing a threshold (dotted vertical line) above which firing frequency jumps to the hyper-excitable, bursting regime. Prior to ramp onset (data not shown) and low levels of autapse strength stellate cells fire at baseline, DC current dependent frequencies. At a critical level of autapse strength (0.8 nS in this example) a small further increase in autapse conductance causes a sudden transition to the high frequency firing regime consistent with our modeling work. **B**: Instantaneous frequency jumps rapidly with increasing tonic current in autaptically coupled SCs. A control stellate cell (not coupled with an autapse) is given DC current steps increasing in magnitude. Firing rate increases gradually and continuously as a function of input current (right axis). When the same cell is coupled with an autapse of constant strength firing frequency undergoes a rapid transition to the hyper-excitable regime when identical current steps are presented (left axis, note difference in scale). **C**: Linopirdine increases autapse-induced burst duration. Representative example of an autapse experiment. After a period of baseline firing was observed (first panel), the autpase was turned on inducing hyper-excitable, burst firing (second panel). A magnified view of a single burst is seen in the last panel. **D**: After 10 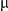
M linopirdine was washed on the same experiment was done on the same cell using the same autapse magnitude. Baseline, uncoupled firing remained qualitatively unchanged (first panel). However, once the autapse is turned on and burst firing is induced, significantly more spikes per burst are observed. The second panel shows a single burst magnified in the last panel to show individual spikes. **E**: M-current blockade with linopirdine significantly increases number of spikes per burst. For all recorded autapse experiments we observed no significant difference between burst ISIs with and without linopirdine application (left bar graph, n = 6). However, burst duration measured as the number of spikes per burst increased significantly (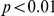) with linopirdine application (right bar graph, n = 6). Error bars are SEM. Linopirdine alters the shape of the spike after hyperpolarization on a fast time scale.

To confirm the predictions of our theoretical results and to show that they are not a artifact of the model, we performed an experimental investigation of the role of recurrent excitation on hyper-excitable firing in *in vitro* mEC SCs. To make experimental investigation tractable and to allow the comparison between our theoretical and experimental results we proceeded as above and approximated a network of recurrently coupled SCs with a single self-coupled SC. In all experiments SCs were synaptically isolated from surrounding cells with AMPA and GABA_*A*_ blockers (see Methods). We used dynamic clamp to mimic autapses onto patch-clamped SCs and asked whether small increases in the strength of the autapse could produce hyper-excitable firing as observed in our simulation results (Figures 1 to 3). We also asked whether *in vitro* self-coupled SCs undergo abrupt, threshold-like transitions to the hyper-excitable regime. To start, SCs were depolarized with constant current to achieve a spike rate of about 2 Hz on average (4–6 Hz spike clusters separated by longer inter-cluster intervals). Over the course of each experiment, autapse maximal conductance 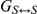 was increased in a ramp-like manner from 0–6 nS. In all cells tested using this protocol, we observed a transition from regular spiking to high frequency, burst spiking (representative example, Figure 9-C, first panel control, second and third panels with autapse).

To confirm that the transition to the high frequency firing regime is a threshold-like phenomenon (defined here as disproportionately large change in firing frequency given a small parameter change) in autaptically coupled SCs, we measured the instantaneous frequency (see Methods) of spike trains produced by autapse conductance ramps. Using these protocols, before autapse strength reached it's threshold level, theta frequency (4–6 Hz) activity was observed. Consistent with modeling results (Figures 2-B, -C and 3-B), changes in the strength of self-coupling below the threshold for hyper-excitability did not appear to change the firing frequency to any significant degree.

When autapse strength reached approximately 0.8 nS (Figure 9-A, dashed line) instantaneous firing frequency jumped from the theta range to approximately 60 Hz. Further increases in the autapse strength parameter (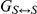) continued to increase the firing rate but no further abrupt transitions in frequency were observed. Thus we conclude that, consistent with modeling results, *in vitro* SCs can undergo abrupt transitions in firing rate due to small increases in self-excitation.

#### The abrupt changes in firing frequency in *in vitro* SCs are the result of phasic but not tonic excitation

Next, we investigated whether *in vitro* SCs could abruptly transition to the high frequency firing regime when given DC current (control) or whether phasic, synaptic current (from an autapse) was necessary. In these experiments, current steps were used instead of current ramps. We found no difference in the qualitative outcome of these two protocols. We applied current steps of increasing magnitude both with an autapse (phasic current) and without an autapse present (tonic current only, control). Figure 9-B shows a representative example in which we compare current steps given to an autaptically coupled cell (black line) to the same cell under control conditions when there was no autapse (gray line). In the control case, the frequency of spikes increased linearly with increasing current and reached a maximal frequency of around 20 Hz when the current steps had a magnitude of around 300 pA (Figure 9-B, gray line, right axis). In all experimental data presented, frequency was computed as the intra-cluster or intra-burst frequency. Clusters and bursts were distinguished as groups of spikes separated by ISIs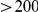 ms. When an autapse was present (constant value of 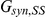 = 10 nS in this example), triggering single spikes due to tonic injected current would immediately trigger an entire train of spikes as the recurrent coupling provided additional positive feedback resulting in continued spiking until adaptation mechanisms activated. At around 100 pA injected DC current, instantaneous frequency (computed from intra-burst intervals only) rapidly jumped to about 150 Hz and remained there for the duration of the experiment. Firing frequency did not change significantly for higher values of applied current. This same behavior was seen in all cells tested (n = 6). We conclude that abrupt, threshold-like transitions in firing rate can occur in recurrently coupled SCs with small increases in phasic current but not isolated (uncoupled) unstellate cells. These experimental results are in good qualitative agreement with our modeling results (Figure 3).

#### Blockade of the M-current in autapse-coupled, *in vitro* SCs increases the number of spikes per burst

Both modeling and experimental results show the existence of a hyper-excitable firing frequency regime. However, there is a difference between the predicted high frequency firing patterns and the experimentally observed ones. The former are persistent (see Figure 1) while the latter are burst-like. This behavior indicates the presence of a slow, adapting mechanism possibly produced by an M-current [49]. The M-current is a slowly activating, depolarization activated and hyperpolarizing current mediated by Kv7 type potassium channels. It is known that blockade of the M-current can cause bursting behavior in hippocampal neurons [50] and some non-stellate EC cells [48] in response to square current pulses. On the other hand, blockade of the M-current has been shown to have no effect on the subthreshold oscillatory activity in SCs oscillatory activity [48].

We build on these studies by investigating the M-current's effect on SC bursting in response to realistic synaptic inputs generated with dynamic clamp. Figures 9-C and -D are representative traces from the same cell being given an identical autapse. Under control conditions (Figure 9-C) SCs fire in proportion to their applied current, 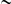
2 Hz in this example (left panel). As with our previous data, all frequency measurements represent intra-cluster or intra-burst frequency. When an autapse is added, burst firing due to self-excitation is induced (Figure 9-C, panel 2). The dotted box in this panel is magnified in the last panel. We note that the representative burst shown has three spikes and then terminates. We then applied 10
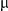
M of the Kv7 channel blocker linopirdine to the same cell and performed the same recording (Figure 9-D). Baseline firing, shown in the first panel, appeared unaltered which is consistent with previous work [48]. Introduction of an autapse, shown in panel 2, induced burst firing as in the control case. However, a magnified burst (Figure 9-D, panel 3) shows that the burst duration increased to around 10 spikes. Thus we conclude that the M-current in SCs is activated at voltages above threshold, during high frequency firing, and modulates burst duration in response to recurrent excitation.

#### Burst duration but not burst interspike interval (ISI) is modulated by the M-current in *in vitro* SCs

To quantify the changes to burst properties we performed the same experiment shown in Figure 9-C, -D for six cells and observed burst durations with and without M-current blockade. To automatically identify the gaps between bursts we searched the data for inter-spike intervals longer than 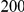 ms. Averaging over all cells showed that burst duration increased significantly (Figure 9-E, right bar plot, p
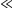
0.01, n = 6) with average burst duration changing from about 3.5 spikes per burst in control (gray bar) to about 7.5 spikes per burst with M-current block (black bar). Interestingly, the intra-burst frequency (the frequency of spikes within a burst) didn't change significantly, as seen in the left bar plot of Figure 9-E and remained at an ISI of about 7 ms regardless of M-current blockade (n = 6). Thus we conclude that in hyper-excitable stellate cells *in vitro* burst duration but not burst ISI is modulated by the M-current. In all cells tested, firing was not persistent even with M-current block as seen in some versions of the model. See our discussion below.

#### Addition of a M-current to the minimal network model produces burst-like behavior in the hyper-excitable frequency regime

**Figure 10:**
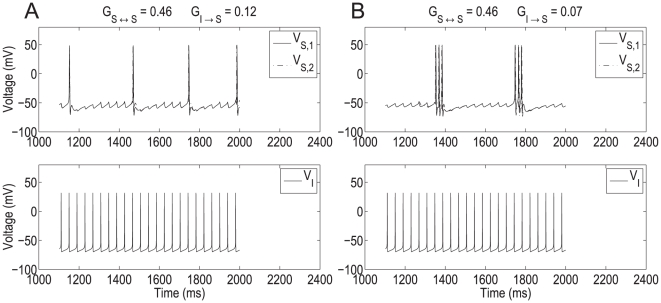
Abrupt transition from the theta to the bursting hyper-excitable regime in a SI minimal network in a stellate cell model including a M-current. The parameters used are 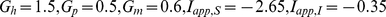 and 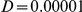.

Consistent with our own and previous experimental findings [48], the parameters corresponding to 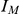 were set so that its blockade did not affect subthreshold oscillatory patterns; i.e., 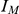 was set to activate for voltage values slightly above the subthreshold regime in contrast to previous studies [8] and to layer V pyramidal cell models [51]. Figures 10-A and -B show the transition from the theta regime (Figure 10-A) to the hyper-excitable regime (Figure 10-B) for the minimal network investigated above (Figure 1-A) when the strength of inhibition (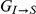) is lowered (Figure 10-B) to mimic the drop in SC inhibitory inputs seen in epileptic animal models. For the larger value of 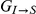 (Figure 10-A), SCs fire in phase at 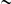
4 Hz (theta regime). For the lower value of 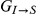 (Figure 10-B), SCs fire bursts of spikes with an interspike interval of 17 ms (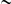
60 Hz) and slightly out of phase with one another. The length of the hyper-excitable burst is modulated by the amount of 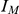 (data not shown).

A qualitatively similar transition from the theta to the burst-like hyper-excitable regime is observed in the self-coupled SC model as the maximal synaptic conductance 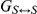 increases above its threshold level (see Figure S7-B). The high-frequency regime is burst-like but the intra-burst interspike-interval remains almost unchanged. Figure S7-B shows the abrupt transition between firing frequency regimes for 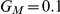. The 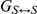 threshold for the transition is not affected by the presence of 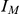. Near this threshold values the number intra-burst spikes is low and increases with 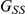 (Figure S7-B top-left to bottom-right panels). The interspike-interval within a burst is almost constant. For a fixed value of 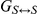 the number of spikes per burst decreases with increasing values of 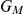 (not shown). In contrast to experimental findings, complete blockade of 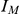 produces persistent firing (Figures 2 and S7-A) indicating that SCs contain additional mechanisms not considered in our model that terminate bursts. We discuss these mechanisms further below.

## Discussion

Previous work has established that stellate cells (SCs) from layer II of the medial entorhinal cortex *in vitro* display rhythmic patterns of voltage activity in the theta frequency regime (4–10 Hz) [4], [12]. These patterns, consisting of subthreshold oscillations and spikes, have been shown to be an intrinsic cell phenomenon [12] resulting from the interaction between the h- (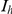) and persistent sodium (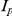) currents [4]. *In vivo* spiking patterns have been shown to be similarly rhythmic in the theta range [13], although there is some question about the link between subthreshold oscillations and *in vivo* firing [14]. Modeling work has contributed to the understanding of the mechanism of generation of theta rhythmic subthreshold oscillations and spiking patterns [4], [9]–[11], [33] in single SCs, and in small networks including SCs and interneurons [8], [10], [11], [37].

Recent studies report that SCs are hyper-excitable in epileptic-like, pilocarpine-treated rats [31], [32] where reduced levels of recurrent inhibition and similar levels of recurrent excitation have been observed in diseased animals as compared to control ones. Similar results to those observed in epileptic animals were shown to occur in control animals under GABA_*A*_ receptor blockade [31]. Furthermore, hyper-excitability in SCs does not appear to be dependent on changes in the intrinsic properties of SCs [23], [32], [52]. These findings raised the question of what biophysical and dynamic mechanisms are responsible for both the generation of the fast time scale corresponding to hyper-excitable firing and the transition between the theta and hyper-excitable firing frequency regimes.

In this paper we have addressed these questions using biophysical (conductance-based) modeling, simulations, dynamical systems tools and dynamic clamp experiments. We have shown that model SCs have the intrinsic dynamic properties that endow them with the ability to fire in the fast, hyper-excitable regime in addition to the theta one. In other words, the fast time scale corresponding to hyper-excitable firing is “built in” the SC as it is the slow time scale corresponding to theta rhythmic activity (generated by the interaction between 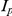 and 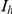). The latter is uncovered with the solely presence of appropriate levels of tonic (constant) drive while the former requires phasic excitation to be uncovered and is “hidden” in control cases. The transition between these two modes of operation is abrupt mostly due to the fast-slow nature of the dynamical system governing the dynamics of the SC model in the subthreshold regime, and it does not require changes in the intrinsic properties of SCs.

In a single, self-connected SC, AMPA recurrent excitation arrives at the cell immediately after a spike has occurred and causes an imbalance between 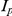 (depolarization activated) and 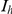 (depolarization deactivated) that favors spiking. Spiking occurs within a short interval after the previous spike provided the amount of recurrent AMPA excitation is large enough for this current imbalance to be maintained for the necessary amount of time. This requires the maximal synaptic conductance and the decay time of excitation to be large enough so the SC's recovery from excitation is slow enough. For combinations of these two parameters such that the amount of AMPA current is lower, the current balance between 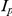 and 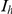 that opposes fast spiking is recovered fast enough, before a new spike occurs, and the SC does not fire again until after roughly a theta cycle.

To show that our findings are not an artifact of the model we performed complementary *in vitro* experiments where real SCs where synaptically self-excited using dynamic clamp. We first showed that *in vitro* stellate cells do indeed exhibit and abrupt transition to fast spiking. Next, we showed a difference in response to depolarizing current steps when a stellate cell was autaptically coupled and when it was not. In the presence of the autapse, the cells jumped abruptly to very high frequency firing, while in its absence the cells proportionally increased their firing rates with the level of injected current.

Firing patterns displayed in SCs were burst-like meaning that periods of high frequency firing triggered by positive feedback were interspersed by periods of quiescence. They resemble those observed in the *in vitro* experiments in [32] in response to brief current pulses in both pilocarpine-treated animals and control animals under GABA_*A*_ receptor blockade. Next, we showed that burst-like firing in the *in vitro* preparation is modulated by the depolarization activated M-current. Addition of an M-current to our model SC resulted in similar burst-firing behavior. Supplemental data (see Supporting Text S1) shows that washing on linopirdine, an M-current blocker, enhances the depolarizing after potential (DAP) in autaptically coupled SCs, an event on the same timescale as monosynaptic inputs (Figure S8). We hypothesize that the amplification of the DAP causes increased receptivity to fast timescale spike generating synaptic inputs lengthening. It has been previously reported that a similar transient spike probability change controls patterned spiking in a stellate cell model with stochastically gating channels [9]. An outstanding difference between our modeling and experimental results is that experimental blockade of the M-current did not produce persistent firing as in our model. This indicates that other currents, not considered in this study, are also involved in the termination of the high frequency bursts. Further research will be required to identify these currents. Possible mechanisms include (i) other potassium currents mediated by Kv2.1 subclass channels which are also (like Kv7) non-inactivating and have slow kinetics, and are not blocked by linopiridine, (ii) fast firing would causing calcium build up and eventually leading to the activation and opening of calcium dependent potassium channels, (iii) sodium depletion after many spikes which could eventually lead to the termination of spiking. In addition, we cannot rule out the possibility that a single-compartment model is too simple to account for the whole phenomenon. However, as our results emphasize, the mechanism of burst termination is independent of the mechanism of transition from “normal” to hyper-excitable firing which is the main focus of this work.

It is well established that the SC model gives highly irregular firing (including clustering) in the theta regime when appropriate levels of noise are added [8], [35], [53]. (See also [33] for a mechanistic description in a similar model). We illustrate irregular firing in the SC model in the theta regime in Figure S9. Adding realistic levels of noise also generate irregular firing in the hyper-excitable regime. In the presence of the M-current, the firing frequency distribution is bimodal (see Figures S10 and S11) with one cluster of firing frequencies in the theta regime and another cluster in the hyper-excitable regime.

In this paper, we did not include the effects of inhibition or the M-current on the phase-space analysis. Previous work [48] by other authors and our experiments have shown that the M-current has a negligible effect on the generation of subthreshold oscillations in the theta frequency regime. Thus, we assumed the M-current is not active in the subthreshold regime. Understanding the M-current's effect on burst duration requires analysis of a separate regime (intermediate between the spiking and subthreshold ones). In principle, 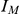 opposses the effects of synaptic excitation. A building-up mechanism for 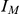 as the result of succesive pulses of synaptic excitation is a plausible candidate that could explain the fact that 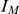 modulates the length of the bursts while the intra-burst frequency remains almost unchanged.

The results for single, self-connected SCs have implications for networks of recurrently connected SCs. However, the mechanism is not straightforward. Unlike single, self-connected cells, in large networks each cell contributes a low amount of AMPA excitation and there is no reason to believe that most inputs coming from other cells arrive within the window opportunity for fast spiking referred to above. In these networks, SCs first need to fire close enough, an ability demonstrated theoretically and experimentally (*in vitro*) in [8], [54]. For each SC, the transition to fast spiking occurs once the amount of recurrent excitation received right after a spike has occurred reaches a “critical mass”. All SCs in the network are expected to be recruited to fast spiking after a few theta cycles. Our results on networks of two SCs transitioning to fast spiking makes the generation of out-of-phase clusters at theta frequencies very implausible. We hypothesize that the anatomically realistic recurrent coupling, seen in epileptic animal models [32], promotes firing on the fast timescale.

Our results for recurrently connected networks of SCs receiving synaptic inhibition from interneurons show that additional transitions between firing frequency regimes occur depending on the level of inhibition onto SCs. Inhibition acts as an external switch: it is not necessary for the generation of the hyper-excitable firing regime. Instead, inhibition serves to modulate the cell's firing frequency regime. The fact that synaptic inhibition above some threshold value helps maintain the current balance necessary for firing at theta frequencies is not surprising since synaptic inhibition acts by opposing the effects of synaptic excitation. However, synaptic inhibition promotes firing as the result of its interaction with the h-current [38]. Whether this switching mechanism is at work in larger networks of recurrently connected SCs remains an open question. Synaptic inhibition has a dual effect on SCs. It both promots and disrupt synchronization in phase [10]. In the latter case, it would be preventing recruitment of SCs into fast spiking. Our results explaining neural excitability due to the interaction between intrinsic and network properties complement previous studies [36], [43]–[46].

Reduction of inhibition and recurrent connectivity between SCs has been observed in epileptic tissue [31] *in vitro*. The present work provides insights into the implications of this type of network arrangement for the generation of hyper-excitable firing. While the hyper-excitable firing patterns shown in this study do not represent the full range of firing behaviors seen in a true epileptic seizure our results explain in great mechanistic detail how physiologically realistic network topology can lead to hyper-excitable firing. Our simulations demonstrate that the rapid, threshold like transition to the high frequency firing regime does not require changes in the intrinsic properties of the SC, and is modulated by network inhibition, recurrent excitation and intrinsic biophysical mechanisms. Our experiments (see Supporting Text S1 and Figure S8) show the timing of rapid synaptic feedback is similar to that of SC DAPs which are amplified by M-current block implying a role for the M-current in preventing high frequency neural discharges. This finding suggests a possible method of action for retigabine, an M-current enhancer currently under investigation for therapeutic use for its anti-convulsant properties [55]. Our results suggest that a drug such as retigabine would decrease burst duration by making fast time scale spike generation more difficult. Further research is warranted to investigate the effect of retigabine on the SC DAP and high frequency spiking behavior. The present study provides evidence that stellate cells naturally contain two timescales for spike output. The slow timescale is associated with theta frequency spiking while the fast timescale depends on low levels of inhibition and recurrent excitation and produces hyper-excitable firing as observed in epileptic animals.

## Materials and Methods

### Modeling: Biophysical models for stellate cells, interneurons and synaptic connections

We use single compartment biophysical (conductance-based) models for both stellate cells (SCs) and interneurons (inhibitory cells or ICs) and consider synaptically coupled networks of these cells. For SCs we use the model introduced in [8], which is based on measurements from layer II SCs of the medial entorhinal cortex (MEC) [4], [33]–[35]. It has the following currents: transient sodium (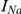), delayed rectifier potassium (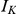), leak (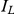), persistent sodium (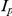), a two-component (fast and slow) hyperpolarization-activated (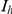 or h-) current, and a slow potassium (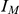 or M-) current. The current-balance equation for single SCs is
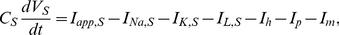
(7)where 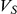 is the membrane potential (mV), 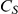 is the membrane capacitance (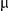
F/cm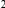), 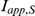 is the applied bias (DC) current (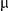
A/cm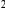), 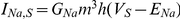, 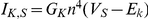, 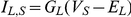, 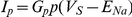, 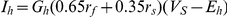, and 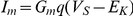. For interneurons (inhibitory cells or ICs) we use the biophysical model introduced in [56]. The current-balance equation for single ICs is
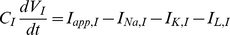
(8)where 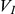 is the membrane potential (mV), 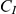 is the membrane capacitance (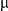
F/cm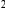), 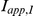 is the applied bias (DC) current (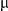
A/cm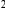), 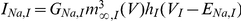, 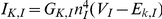, 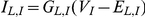.

For both cell types 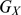 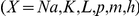 and 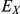 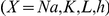 are the maximal conductances (mS/cm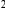) and reversal potentials (mV) respectively. The units of time are msec. All the gating variables 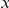 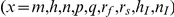 obey a first order differential equation of the following form:
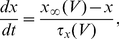
(9)where the activation/inactivation curves and voltage-dependent time scales are given respectively by
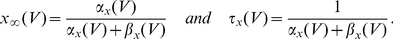
(10)


For the gating variables corresponding to the I-cell (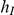 and 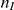) the right hand side of eq. (9) is multiplied by a parameter 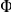 = 5 [56]. The definitions of the functions in eq. (10) for all the gating variables are given below. Graphs of 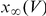 and 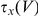 for the gating variables 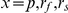 are presented in Figure S4. For the remaining gating variables, the graphs of 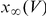 and 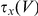 are standard. We use the following parameter values [8], [56]: 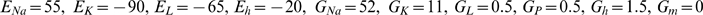, 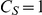, 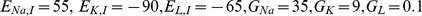 and 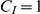. In our simulation we keep all parameters fixed except for 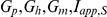 and the maximal synaptic conductances that we describe below.

For networks we add synaptic connectivity terms of the form
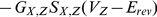
(11)to the current balance equations (7) and (8), where X and Z represent the presynaptic and postsynaptic cell respectively with 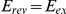 or 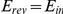 (mV) according to whether the presynaptic (X) cell is excitatory or inhibitory respectively. The parameters 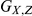 are the maximal synaptic conductances (mS/cm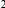). The excitatory and inhibitory reversal potentials are 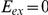 and 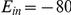 respectively. The synaptic variables 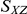 obey the following kinetic equations
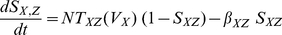
(12)where
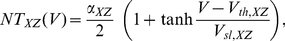
(13)and 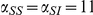, 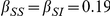, 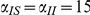, 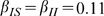, 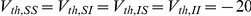 and 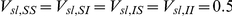.

Following [35] we introduce channel white noise in the persistent sodium current (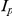) in some of our simulations. More specifically, we add a stochastic term 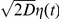 to the dynamic equation for the gating variable 
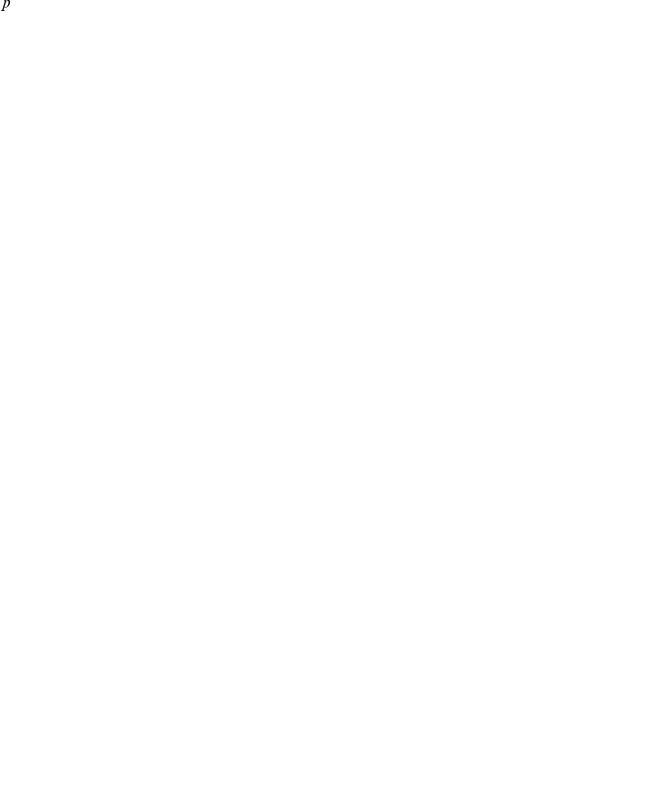
. This term is delta correlated with zero mean; i.e., 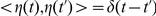. A similar stochastic formulation has been used to generate subthreshold oscillations (and spikes) in SCs in an otherwise silent SC [10].

The functions 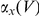 and 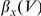 used in the definitions of 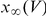 and 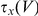 or, in some cases, the latter functions are given by
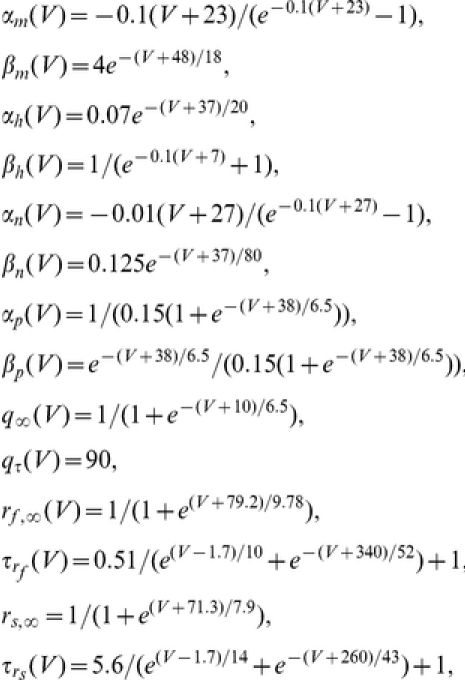


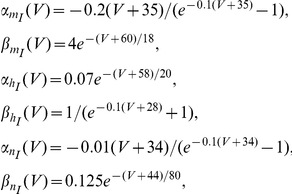



Note that 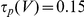 and 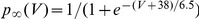.

Simulations were performed using XPP [57] (using parameters: meth = cvode, toler = 1e-5, dt = 0.02) and the modified Euler method [58] coded in C. Graphs were generated with MATLAB (The Mathworks, Natick, MA).

### The reduced nonlinear artificially spiking stellate cell (NAS-SC) model

In previous work [10], [11] we studied the mechanism of generation of subthreshold oscillations and the onset of spikes in the SC model (7) (in the absence of 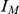). Using dimensionality reduction methods we uncovered a three-dimensional model that captures the dynamics of the SC in the subthreshold regime [10] where subthreshold oscillations are generated and the onset of spikes occur, and provides a good approximation to the “full” SC model in that regime. This reduced 3D model describes the evolution of the membrane potential V and the two h-current gating variables 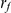 and 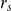 (fast and slow respectively). Notably, 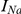 and 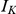 were found to have a negligible effect on subthreshold dynamics and thus, were omitted from the reduced model [10]. The persistent sodium gating variable 
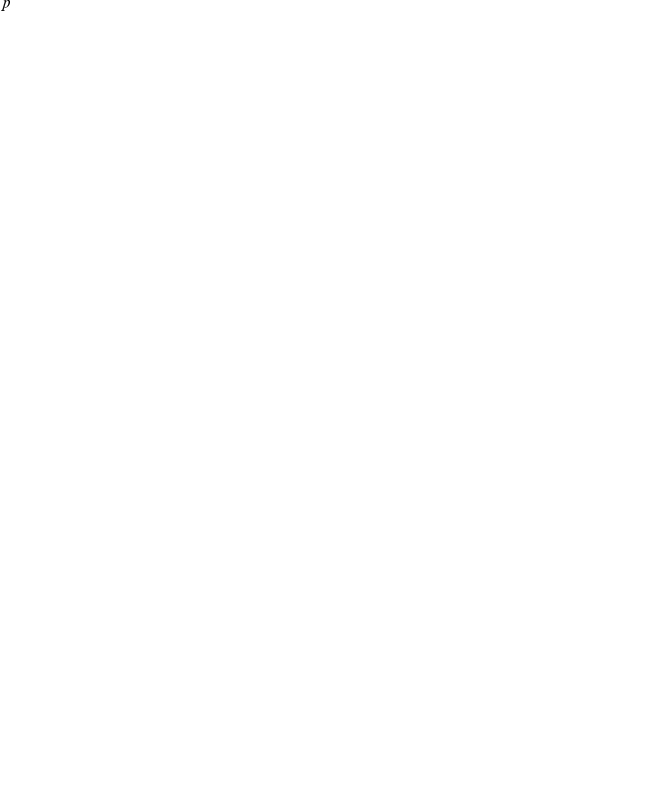
 evolves much faster than the remaining gating variables and the adiabatic approximation 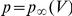 can be made. The resulting equations are
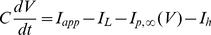
(14)

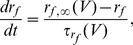
(15)

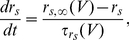
(16)where 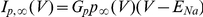. Equations (14)–(16) describe the dynamics of the SC in subthreshold regime, including both the generation of subthreshold oscillations and the onset of spikes [10], [11], but they do not describe the spike dynamics which belong in a different regime (where 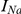 and 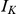 are the main active currents).

If spiking dynamics are not of interest but only the occurrence of spikes, the dynamics of a SC can be approximately described by Eqs. (14)–(16) supplemented with a zero width “artificial spike” (occurring on a short time scale and reaching a peak of about 60mV) and an appropriate threshold (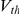) and reset (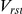) values. This reduced model was termed the Nonlinear Artificially Spiking SC (NAS-SC) model [10]. It is a class of model that includes the generalized integrate-and-fire and resonate-and-fire models [59], [60]. As we describe later, in our NAS model the onset of a spike occurs when the voltage begins to evolve on a fast time scale as a consequence of the cell's dynamics, and without bounds, in the subthreshold regime. This indicates that the voltage is “escaping” the subthreshold regime and entering the spiking one. We choose 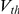 such that it lies close to the end of the subthreshold regime. Once the trajectory reaches the threshold value the voltage is reset to its initial, subthreshold value. Note that in contrast to other models, crossing 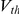 is not part of the mechanism of spike generation and only indicates spike occurrence [10]. The onset of spikes, however, is accurately described by Eqs. (14)–(16). The reset values 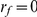 and 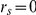 can be derived from the seven-dimensional SC model [10], [11]. The reset value 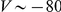 mV is an estimate from our numerical simulations. We take 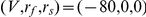 as the initial conditions in the subthreshold regime, and we reset the trajectory to these values after each spike has occurred.

In the full SC model there is a brief intermediate regime in between the spiking and the subthreshold regimes [10]. This intermediate regime corresponds to the recovery of the voltage after a spike or AHP. It is different from the subthreshold regime in that 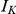 is not negligible and its corresponding gating variable n evolves on a time scale faster than both rf and rs. In this regime, both 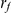 and 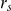 evolve slowly (and for a short amount of time) from their reset values 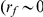 and 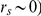 to slightly higher ones. In the NAS-SC model approximation we are neglecting both the intermediate and spiking regimes, and we are setting the initial conditions at 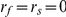. The patterns obtained using the NAS-SC and the full SC models are qualitatively similar. However, quantitative comparable results can be obtained by adjusting some parameters such as 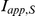 or the maximal conductances.

#### Dimensionless formulation

Here we bring system (14–16) to a dimensionless form in order to uncover the different time scales in which the system operates. We first choose appropriate voltage and time reference scales, 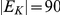 mV and 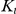 respectively, and define
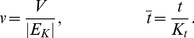
(17)


Note that the spiking cell's voltage range is bounded by the spiking currents reversal potentials 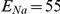 mV and 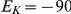 mV; so the dimensionless voltage 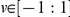. A relevant voltage range for the subthreshold regime, in terms of the dimensionless variable 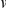, is 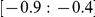 [10], [11]. We define
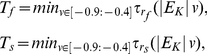
(18)and we choose 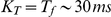 (see Figure S4-B). We also choose a reference maximal conductance 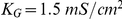, which is at the top of the range of physiologically plausible values of 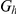 we use in our simulations. We define the following dimensionless variables, parameters and functions
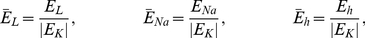
(19)

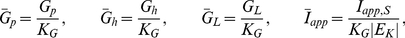
(20)

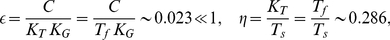
(21)

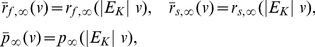
(22)

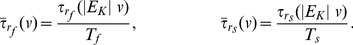
(23)


Substituting eqs. (17–23) into eqs. (14–16) and deleting the “bar” sign yields
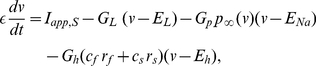
(24)

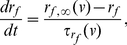
(25)

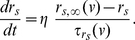
(26)


System (24)–(26) is a fast-slow system with 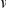 evolving on the fast time scale and both 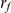 and 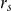 evolving on a slow scale. These two variables evolve on a similar slow time-scale, as it becomes apparent by comparing the values of 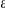 and 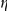 (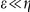). The dimensionless voltage threshold and reset values are: 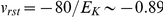 and 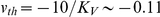.

System (24)–(26) describes the slow dynamics. The fast dynamics is described by the following system obtained by a further rescaling 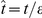:
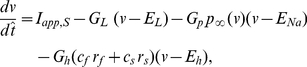
(27)

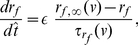
(28)

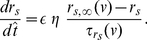
(29)


### Experimental

#### Brain slice and electrophysiology

All protocols were approved by the Institutional Animal Use and Care Committees at Boston University (approval #07-014) and University of Utah (approval #07-09009). Experimental protocols were the same at both locations. Brain slices were prepared using established techniques [54]. Briefly, young, Long-Evans rats (postnatal days 18–28) were anesthetized using isoflurane and decapitated. The brain was removed rapidly and placed in a beaker of ice cold, oxygenated artificial cerebrospinal fluid (ACSF) containing (in mM): NaCl 124, KCl 5, KH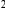, PO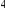 1.2, CaCl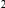 2.4, MgSO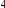 2.6, NaHCO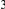 26, D-glucose 25. The pH was maintained at 7.4 by saturation with 95% O2–5% CO2. The brain was dissected down to a block of tissue containing the region of interest. A Vibratome (TPI) was used to cut slices, 350–450
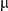
m thick in the horizontal plane. Slices were incubated at 32

C for at least 30 min and 30 min at room temperature before use.

Entorhinal cells were identified under differential interference contrast (DIC) optics based on morphology and location, then patched with 4–6 M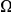 glass pipettes filled with internal solution that contained (in mM): 120 K-Gluconate, 10 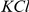, 10 HEPES, 4 Mg-ATP, 0.3 Tris-GTP, 10 Na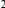-Phosphocreatine, 20 U/ml Creatine Kinase. Pipettes were connected to the headstage of an amplifier (MultiClamp 700B) set in “bridge” mode to obtain current clamp recordings. Input resistance, resting potential, and capacitance compensation settings were continually monitored. Stellate cells in the medial entorhinal cortex were identified using established criteria [61] such as the presence of the hyperpolarization activated cation current Ih, spike clustering and peri-threshold oscillations.

#### Dynamic clamp

A real-time dynamic clamp system [62] was used to mimic the recurrent connections between a population of stellate cells. “Autapses” were modeled with a double exponential function with rise time constant of 0.3 ms and fall time constant of 5.6 ms [63]. The sum of the synaptic delay and conduction delay was modeled to be 3 ms. Synaptic currents obeyed the equation 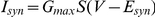 where 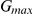 is the maximal synaptic conductance, 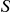 is the synaptic variable, 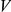 is the post-synaptic voltage supplied by the dynamic clamp and 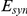 is the synaptic reversal potential which was set to 0 mV. After cells were patch clamped, their membrane voltage was supplied to the dynamic clamp which detected, in real time, the occurrence of spikes in the voltage trace. After a spike was detected and the synaptic delay had passed the dynamic clamp computed the time varying, voltage dependent output current necessary to mimic the desired synaptic waveform.

#### Analysis

Dynamic clamp data was analyzed with MATLAB (The Mathworks, Natick, MA). Dynamic clamp protocols were written to enable constant value autapses and autapse ramps. Frequency vs. 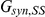 plots were computed from autapse ramps. Burst spike frequency was computed from instantaneous ISIs during bursts only. Bursts were differentiated by the relatively long ISIs that separated them. Stellate cells tend to cluster their spikes under control conditons and spike frequency was computed from intra-cluster ISIs only. Clusters were automatically identified as any group of spikes separated by an ISI larger than 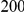 ms. The same method was used to identify bursts. Frequency-current plots were made from the spike frequency measurements described above. Once burst spikes had been counted these numbers were averaged over multiple trials and significance computed with a one way ANOVA. AHP statistics were computed from spontaneous spiking when no autapse was present and was averaged over all detected spikes. Plots were produced in MATLAB and finalized with the Inkscape graphics editor (inkscape.org).

## Supporting Information

Text S1 The stellate cells depolarizing afterpotential (DAP) is on the same time scale as the ISI of burst spikes. This implies that hyperexcitable firing may be facilitated by the presence of a DAP and that enhancements of the DAP, such as an autapse, would serve to enhance hyperexcitable firing behavior.
(0.03 MB ZIP)

Figure S1 Activation/deactivation curves (x1_∞_(V)) and voltage-dependent time scales (τ_x_(V)) for I_p_ (x = p) and I_h_ (x = rf , rs) in the stellate cell model (1)–(3). The bottom panels are magnifications of the top ones.
(0.11 MB EPS)

Figure S2 Threshold values of G_S⇔S_ as a function of I_app,S_ (top panels) and G_h_ (bottom panels) for the SC model (7D) for two values of the isolated SC's firing frequency: (a) ∼3 Hz (3 spikes/sec), (b) ∼4 Hz (4 spikes per sec). Points in parameter space below and above the lines correspond to theta and fast firing frequency respectively. The values of I_app,S_ and G_h_ corresponding to the same critical value of G_S⇔S_ obey the quasi-linear curve shown in Fig. S3 to maintain a constant natural frequency for the isolated SC.
(0.07 MB EPS)

Figure S3 Curves of constant values of firing frequency for the (7D) SC model. The four higher firing frequencies (6 to 12 Hz) have been calculated as the reciprocal of the interspike interval. The lower two firing frequencies (3 and 4 Hz) have been approximated by the number of spikes per second. In the latter cases, the curves shown correspond to regular patterns displaying two and one subthreshold oscillations per spike respectively. The interspike interval slightly increases as the value of I_app,S_ increases. (The M-current I_m_ has not been included in these simulations.)
(0.05 MB EPS)

Figure S4 Voltage traces and phase-space diagram for the NAS-SC model in the theta regime (slow time scale). The value of I_app,S_ = −2.35 is larger than the one corresponding to Fig. 7. The values of the other parameters are the same (G_h_ = 1.5, G_p_ = 0.5) as in Fig. 7. The bottom panels are a magnification of the top ones. (a) Voltage trace showing two subthreshold oscillations. (b) Two-dimensional representation of the phase-space. See main text for explanations.
(0.22 MB EPS)

Figure S5 Voltage traces (left) and two-dimensional phase-space representations for the NAS-SC model for values of I_app,S_ that increase from Fig. 5-top (this figure) to Fig. 6-bottom (next figure). As the value of I_app,S_ gradually increases, the V-nullsurface gradually moves down, thus facilitating firing; i.e., the firing frequency gradually increases.
(0.34 MB EPS)

Figure S6 Voltage traces (left) and two-dimensional phase-space representations for the NAS-SC model for values of I_app,S_ that increase from Fig. 5-top (previous figure) to Fig. 6-bottom (this figure). As the value of I_app,S_ gradually increases, the V-nullsurface gradually moves down, thus facilitating firing; i.e., the firing frequency gradually increases.
(0.21 MB EPS)

Figure S7 Effects of IM on hyper-excitability in single SCs self-connected with an autapse. (a) Persistent spiking patterns in the hyper-excitable firing regime in the absence of I_m_. (b) Bursting spiking patterns in the hyper-excitable regime in the presence of I_m_. The number of bursts per spike increases with increasing values of the maximal synaptic conductance. The following parameters were used: G_h_ = 1.5, G_p_ = 0.5 and I_app,S_ = −2.5.
(0.23 MB EPS)

Figure S8 M-current block amplifies the depolarizing afterpotential (DAP) in stellate cells. Voltage traces from all cells used for analysis in panel (a) were scanned for spikes prior to the onset of the autapse (eg. control spiking where G_syn,SS_ = 0). The voltage traces following those spikes were averaged. On a slow time scale (100s of ms) the DAP characteristic of stellate cells was obvious from this average as well as the tendency of stellate cells to cluster their spikes seen as the grouping of spikelets on the peak of the overshoot. Additionally, on a fast time scale (5–10 ms), the brief depolarization seen right after the spike (arrow) was amplified dramatically in the traces from cells treated with linopirdine. We note that this depolarization is on the timescale of the burst ISI.
(0.11 MB EPS)

Figure S9 Irregular spiking pattern in an isolated SC model with persistent sodium noise. (a) Voltage trace. The right panel is a magnification of the left one. (b) firing frequency histogram. The firing frequency r was calculated as the reciprocal of the interspike-interval multiplied by 1000 ms. Parameters used: G_h_ = 1.5, G_p_ = 0.5, G_q_ = 0.5, G_SS_ = 0, D = 0.001. Spiking occurs only in the theta frequency regime.
(0.37 MB EPS)

Figure S10 Irregular spiking pattern in a self-connected SC model with persistent sodium noise. (a) Voltage trace. The right panel is a magnification of the left one. (b) firing frequency histogram. The firing frequency r was calculated as the reciprocal of the interspike-interval multiplied by 1000 ms. Parameters used: G_h_ = 1.5, G_p_ = 0.5, G_q_ = 0.5, G_SS_ = 0.56, D = 0.001. Burst of fast frequency spikes. The intra-burst frequency corresponds to the fast time scale while the inter-burst frequency corresponds to the theta time scale.
(0.38 MB EPS)

Figure S11 Irregular spiking pattern in a self-connected SC model with persistent sodium noise. (a) Voltage trace. The right panel is a magnification of the left one. (b) firing frequency histogram. The firing frequency r was calculated as the reciprocal of the interspike-interval multiplied by 1000 ms. Parameters used: G_h_ = 1.5, G_p_ = 0.5, G_q_ = 0.15, G_SS_ = 0.56, D = 0.001. Burst of fast frequency spikes. The intra-burst frequency corresponds to the fast time scale while the inter-burst frequency corresponds to the theta time scale.
(0.42 MB EPS)
