# Supplementary material for: The Mechanism of Abrupt Transition between Theta and Hyper-Excitable Spiking Activity in Medial Entorhinal Cortex Layer II Stellate Cells
Source: PLoS One. 2010 Nov 4;5(11):e13697. doi: 10.1371/journal.pone.0013697 (PMC2973955; doi:10.1371/journal.pone.0013697)
Supplement: Text S1 — The stellate cells depolarizing afterpotential (DAP) is on the same time scale as the ISI of burst spikes. This implies that hyperexcitable firing may be facilitated by the presence of a DAP and that enhancements of the DAP, such as an autapse, would serve to enhance hyperexcitable firing behavior. (0.03 MB ZIP) [file pone.0013697.s001.zip › SupplementaryTextS1.pdf]

The stellate cells depolarizing afterpotential is on the same time scale as the ISI of burst spikes

Stellate cells (SCs) show a depolarizing afterpotential (DAP) [1, 2] which is a small depolarization following about 5 ms after an action potential. The time scale of this depolarization is on the order of monosynaptic feedback and it has been shown in hippocampal and EC neurons [1, 3] that the DAP becomes more depolarized when the M-current is blocked. Since adding M-current to our model (Fig. 10-F, main manuscript) produces burst firing like that observed in our experiments, M-current block has been shown to cause a depolarizing shift in the SC DAP [1], and the SC DAP is on the timescale of monosynaptic excitation we sought to investigate how these properties of our experimental system were interacting to produce hyper-excitable firing.

Our control recordings in Fig. S8 (gray trace) show the presence of the SC DAP following a spike [2]. Upon application of linopirdine, the DAP was increased in size by approximately 2 mV (Fig. S8, black trace, arrowhead) which is consistent with previously published results [4]. Thus, stellate cells have a short, post-spike time window in which they are depolarized relative to rest. We hypothesize that the two inherent time scales of firing in SCs (normal, theta firing and fast, hyper-excitable firing) correspond to the two most depolarized time windows in the full SC after spike waveform. The shape of the full after spike waveform (Fig. S8) shows that the cell's membrane voltage is most depolarized during the short timescale DAP (arrow) and during the overshoot, approximately 150 ms after the spike. These two depolarizations are on timescales similar to theta and hyper-excitable firing that we have observed in both model and experimental results. M-current blockade further depolarizes the short timescale DAP (Fig. S8, black trace, arrow) implying that spike generation probability would be increased on this timescale. We hypothesize that this increase in spike likelihood during fast time scale activity is related to the increased burst duration we measured (Fig. 9-E, main manuscript). Given our modeling and experimental results showing that monosynaptic self-excitation can lead to hyper-excitable firing in SCs, we consider it possible that this effect is enhanced by the concurrency of synaptic input and a DAP and enhanced further by a more depolarized DAP. Consistent with this hypothesis, our experimental results show that M-current blockade amplifies the DAP and produces lengthened bursts during hyper-excitable firing (Fig. 9-E, main manuscript).

Finally, M-current block, which has been shown to produce an adaptation rate change in many cell types [5] had no significant effect on the average ISI during periods of high frequency firing (Fig. 9-E, left bar plot, main manuscript). In these figures, frequency is computed from intra-burst spikes only. The lack of a change in the ISI in our experiments

would imply that factors other than the M-current are influential and modulating high frequency spike rate. Our observation that the DAP and realistic synaptic input are on the same timescale could mean that the concurrency of these two events is more important for setting the frequency of firing than the presence of an M-current.

References

[1] M. Yoshida and A. Alonso. Cell-type-specific modulation of intrinsic firing properties and subthreshold membrane oscillations by the M(Kv7)-currents in neurons of the entorhinal cortex. *J. Neurophysiol.*, 98:2779–2994, 2007.

[2] R. M. Klink and A. A. Alonso. Ionic mechanisms of muscarinic depolarization in entorhinal cortex layer II neurons. *J. Neurophysiol.*, 77:1829–1843, 1997.

[3] C. Yue and Y. Yaari. KCNQ/M channels control spike afterdepolarization and burst generation in hippocampal neurons. *J. Neurosci.*, 24:4614–1624, 2004.

[4] A. A. Alonso and R. Klink. Differential electroresponsiveness of stellate and pyramidal-like cells of medial entorhinal cortex layer II. *J. Neurophysiol.*, 70:144–157, 1993.

[5] S. P. Aiken, Lampe. B. J., P. A. Murphy, and B. S. Brown. Reduction of spike frequency adaptation and blockade of M-current in rat CA1 pyramidal neurones by linopirdine (DuP996), a neurotransmitter release enhancer. *Br. J. Pharmacol.*, 115:1163–1168, 1995.
